# Factors influencing physical activity for hypertension prevention among older adults in a peri-urban community in Ho, Ghana

**DOI:** 10.1371/journal.pone.0337053

**Published:** 2025-12-15

**Authors:** Kennedy Diema Konlan, Ruth Nimota, Mirialys Fiona Nana Ama Anaman, Anita Fafa Dartey, Elvis Reindolf Kale, Judith A. Anaman-Torgbor

**Affiliations:** 1 Department of Public Health Nursing, School of Nursing and Midwifery, University of Health and Allied Sciences, Ho, Volta Region, Ghana; 2 Department of Preventive Health, School of Nursing and Midwifery, University for Development Studies, Tamale, Northern Region, Ghana; 3 School of Medicine, University of Health and Allied Sciences, Ho, Volta Region,; 4 Ho Teaching Hospital, Ho Volta Region, Ghana; Universidad Peruana Cayetano Heredia Facultad de Medicina, PERU

## Abstract

**Background:**

Regular physical activity is proven to be an effective risk-reduction strategy against hypertension among older adults. This study assessed the factors influencing regular physical activity among older adults in a peri-urban community.

**Methodology:**

A cross-sectional study was conducted among 276 older adults aged 60 years and above, with or without hypertension, in the Ho Municipality. A convenience sampling method was employed for older adults to respond to a self-developed pretested questionnaire. Data was cleaned and analyzed using Statistical Package for Social Sciences (SPSS) and summarized using descriptive statistics, and chi-square tests of association. Variables found to be statistically significant (*p-value* = 0.05) from the univariate analysis were modeled into a regression equation and reported as odds ratios.

**Findings:**

The overall knowledge levels of the participants on regular physical activity showed a majority (57.6%) had low knowledge of regular physical activity, a significant association between the level of education (χ² = 8.518, *p-value* = 0.004), and the number of people in the household (χ² = 8.154, *p-value* = 0.043) with the level of knowledge. The results also showed that the majority (56.2%) of the participants self-reported low levels of regular physical activity. Participants between 60 to 64 years old had 4.740 times the odds of participating in physical activities than those aged above 80 years (AOR = 4.740, 95%CI:1.472–15.257 *p-value* = 0.009). The respondent’s level of education significantly predicted the likelihood of engaging in physical activity (AOR = 1.814, 95%CI:1.024–3.212, *p-value* = 0.041).

**Conclusion:**

The reported low knowledge of physical activity among older adults highlights the importance of health education through leveraging community health workers and volunteers. Understanding specific barriers older adults face in engaging in physical activity, such as health concerns, lack of access to appropriate facilities, and cultural beliefs, may influence the strategic allocation of resources.

## Introduction

Physical activity refers to any bodily movement produced by skeletal muscles that requires energy expenditure [1]. The benefits of physical activity among older adults are enormous. Regular physical activity has been proven to be an effective preventive strategy against diabetes, cardiovascular diseases, and hypertension [2,3]. Physical activity promotes older adults’ health and functional abilities, thereby improving their quality of life [4] and overall cardiovascular health [5,6]. Despite the well-established benefits of physical activity, there exists a noticeable gap in consistent adoption among older adults [7,8]. Physical inactivity is considered the fourth greatest risk factor for mortality, especially among older adults [7,9]. Physical activity encompasses various dimensions such as frequency, duration, intensity, types, context, and purpose. People who did not engage in physical activity had a 20% to 30% greater risk of mortality from any cause than individuals who performed at least 30 minutes of physical activity on most days of the week [10,11]. As people age and become less active, skeletal muscle’s lipoprotein lipase activity decreases, and insulin resistance may increase. These changes often result in chronic diseases such as atherosclerosis and its sequelae, including hypertension, diabetes, coronary heart disease, and cardiovascular disease [12,13]. Hypertension appears to be a precursor to some complications and may result from poor physical activity. Hypertension is high Blood Pressure (BP) 140 mmHg or higher systolic blood pressure and 80 mmHg or higher diastolic blood pressure readings [14]. Hypertension poses a significant health challenge among older adults worldwide, contributing to increased morbidity and mortality rates [15]. The prevalence of hypertension, diabetes, and other cardiovascular diseases appears to be increasing, especially among older adults [16–18]. Older adults facing these chronic conditions or physical limitations often engage in less physical activity due to health-related constraints [19,20], lack of a supportive physical environment [12], unstable economic status [21], inadequate social systems [14,21], and cultural beliefs support low physical activity among older adults [22].

Given that physical inactivity is a critical risk for hypertension [13] and following the increasing knowledge of the diseases among the general population [21,23], older adults ought to adopt it to promote overall health and reduce risk. However, older adults will likely adopt positive physical activity for hypertension prevention if the social and physical environment and community facilities support it [21]. Socioeconomic factors, such as income and access to transportation, spaces, community-level facilities, cultural orientation, social cohesion, and improved lay knowledge, also played a crucial role in the adoption of physical activity [16,21]. Physical inactivity among older adults is further aggravated by the lack of suitable recreational facilities and cultural perspectives influencing participation in fitness programs or attending recreational activities [16–18]. In Ghana, hypertension prevalence is high, at 2.8% to 67.5% [11,24], particularly among older adults, with profound implications for public health [18]. Concurrently, physical inactivity levels among older adults in Ghana are notably low [25]. This is concerning, given the established correlation between low physical activity levels and increased hypertension risk among older adults [11,24,26]. Therefore, identifying the factors that influence physical activity in older adults will aid the development of strategies to mitigate sedentary behavior and promote active lifestyles [27]. This study assessed the factors influencing the adoption of physical activity for hypertension among older adults in a peri-urban community.

## Materials and methods

### Study design

This study used a community-based cross-sectional research design to assess the factors influencing physical activity adoption among older adults. Data was collected at a single point in time, and no follow-up was required.

### Study settings

This study was carried out in the Dome Community in the Ho Municipality of the Volta region of Ghana. The Ho municipality has 12,484 older adults aged 50–59 years, 9,161 aged 60–69 years, 4,169 aged 70–79 years, and 2,414 aged 80 years and above [28]. The Asogli people (predominant in the Ho municipality) have five [5] main divisions, each administered by a divisional chief assisted by a council of elders. The main divisions include the Bankoe, Heve, Ahoe, Dome, and Hliha divisions.

### Study population, sample size, and sampling

The study targeted adults aged 60 years and above living in the Dome community, both with and without hypertension. Older adults are defined as individuals aged 60 years and above (United Nations, 2019). The Finite Population Correction formula was used to adjust the initial sample size for the study by adjusting the margin of error:

$\text{N}\;=\frac{z^{\text{2}}p}{e^{\text{2}}}$Where N was the sample size, z was the standard normal deviation for a given level of significance (α). For a 95% confidence level, α = 0.05, z = 1.96, and e was the adjusted margin of error (e = 0.0578), and p was the estimated proportion of the elderly (p = 60%).

$\text{N}\;=\frac{{\text{1}.\text{9}\text{6}}^{\text{2}}\times (\text{0}.\text{6})\times (\text{1}-\text{0}.\text{6})}{{\text{0}.\text{0}\text{5}\text{7}\text{8}}^{\text{2}}}$, N = 275.97, hence N=276,

A convenience sampling technique was employed to select older adults in the Dome community to respond to a self-developed pretested questionnaire through household visitations by trained research assistants. Research assistants visited every home within the community, and those who met the inclusion criteria and consented to participate were recruited.

### Data collection and analysis

The data was collected by trained research assistants. The research assistants are native Ewe speakers (the native language of the study community), they had a minimum of a bachelor’s degree in nursing, and they received two days of training in the administration of the questionnaire, research ethics, and translation of the questionnaire to local languages. The questionnaire was uploaded on Google Forms, and research assistants aided respondents to complete it by reading each item loudly. It took an average of 15–20 minutes to complete each questionnaire. The data collection was done from June to July 2024.

The questionnaire was self-designed after a review of the literature, and it is divided into four sessions (A-D). The first section (A) elicited the demographic characteristics that included age, sex, marital status, religion, place of worship, ethnicity, occupation, retirement status, minimum level of education, socio-economic status, and the number of people in the household. The second section (B) focused on the level of knowledge about physical activity. This was made up of eight [8] items on the awareness of physical activity and four [4] items on the importance of physical activity. The third section (C) focused on the practices of physical activity (made up of 8 items). Finally, section D comprised twelve [12] items on the challenges that older adults encounter in engaging in physical activity as a hypertension prevention measure. The questions (sections B to D) were scored on a five-point Likert-type scale, and the response options were ‘strongly agree =1, agreed =2, neutral=3, disagreed =4, and strongly disagree =5’. The overall knowledge and overall practice scores were obtained by making a sum score. Those scores below the average scores were deemed low knowledge and low practices, respectively. Those above the average score were high in knowledge and high in practices accordingly.

Descriptive analysis, comprising frequency and proportions, was used in reporting the analyses of categorical variables. A composite score was determined for the overall level of knowledge and practice by summing the variables under each respective subtheme. Chi-square analysis embedded in SPSS was used to test the associations between the independent variables and dependent variables (level of overall knowledge and overall practices of physical activity). Regression analyses were further conducted to predict the impact of independent variables on dependent variables on the adoption of physical activity for the prevention of hypertension.

### Pre-test

The self-designed questionnaire was pre-tested among ten older adults (five men and five women) in the Ahoe community, and these respondents were excluded from the main study. The community was selected due to its representative demographic profile and accessibility. The Dome community, which is the study area, shares boundaries with Ahoe community and has almost the same demographic and cultural characteristics. The internal consistency of the instrument was evaluated using Cronbach’s alpha. The results were as follows: 0.9 for the knowledge levels and 0.7 for the practices of physical activity.

### Ethical considerations

Ethical clearance was obtained from the Research Ethics Committee of the Institute of Health Research in the University of Health and Allied Sciences [UHAS REC A8 [89] 23–24]. The study was done according to the guidelines stipulated by the institutional ethics committee and the Declaration of Helsinki on human subject research. Both written and verbal consent were obtained. There was no remuneration, incentives, or inducements given to those who responded to the questionnaire

## Results

### Demographic data

The majority (93.8%) of the older adults were 60–79 years old males (51.8%), single (64.5%), retired (71.0%), and belonged to low to moderate socio-economic class (90.9%). Also, the majority of them had completed basic education (73.9%). Among the older adults who were retired, some worked as traders (15.2%), farmers (16.3%), retired public servants (21.7%), and self-employed (19.2%). The number of people in the household was those living alone (29.3%), 1–3 (59.4%), 4–6 (10.5%), and 7–10 (0.7%). The participants were from the Ewes ethnic group (68.5%). Table 1 shows the demographic characteristics of the respondents.

**Table 1:** Socio-demographic data.

Variables	Frequencies	Percentages (%)
**Age**		
60-79 years	259	93.8%
80-100 years	17	6.2%
**Sex**		
Male	143	51.8%
Female	133	48.2%
**Marital status**		
Not married	178	64.5%
Married	98	35.5%
**Religious Affiliations**		
mono atheist	231	83.7%
poly atheist	45	16.3%
**Are you currently working**		
Yes	80	29.0%
No	196	71.0%
**Socio-Economic Status**		
Low to moderate income	251	90.9
High income	25	9.1

### Factors associated with knowledge of physical activity to reduce hypertension risk

The overall level of knowledge about physical activity for preventing hypertension was low (57.6%). A cross-tabulation was conducted including age, sex, marital status, religion, place of worship, ethnicity, occupation, work before retirement, minimum level of education, socioeconomic status, and number of people in the household against knowledge levels on physical activity. The results showed a significant association between the level of education (χ² = 8.518, *p-value* = 0.004) and the number of people in a household (χ² = 8.154, *p-value* = 0.043). In contrast, there was no significant association between age, sex, marital status, religion, place of worship, ethnicity, occupation, work before retirement, and socioeconomic status with the outcome variable. The analysis revealed a significant association between a minimum level of education and knowledge of physical activity as a hypertension risk prevention strategy. The were higher odds of an older adult with a lower level of education having high knowledge (AOR = 2.32, 95%CI: 1.289–4.170, *p-value* = 0.005) on physical activity to reduce high blood pressure. Table 2 shows the association between knowledge level and socio-demographic characteristics.

**Table 2:** Association between knowledge levels and socio-demographic variables.

	Levels of Knowledge		
Variables	Low	High	χ*² (p-value)*
**Age**			
60-79	152 (95.6%)	107 (91.5%)	2.003 (0.157)
80-100	7 (4.4%)	10 (8.5%)	
**Sex**			
male	78(45.3%)	65(55.6%)	12.654(0.09)
female	94(54.7%)	52(44.4%)	
**Education level**			
Basic	107(67.3%)	97(82.9%)	21.567(0.062)
Above basic education	52(32.7%)	20(17.1%0	
**Marital status**			
Not married	110(69.2%)	68(58.1%)	8.234(0.172)
married	49(30.8%)	49(41.9%)	
**Ethnicity**			
Ga, akan	45(28.3%)	42((35.9%)	7.567(0.865)
Ewe, mole dagbani	114(71.7%)	75(64.1%)	
**Currently employed**			
yes	41(25.8%)	39(33.3%)	9.654(0.765)
no	118(74.2%)	78(66.7%)	
**Religions affiliation**			
Constructed place of worship			
Natural place of worship	134(84.3%)	97(82.9%)	4.023(0.356)
**Work Before Retirement**	25((15.7%)	20(17.1%)	
Not retired (Regularized post-retirement work)	39 (51.3%)	37 (48.7%)	2.443 (0.655)
Trader	25 (59.5%)	17 (40.5%)	
Farmer	25 (55.6%)	20 (44.4%)	
Public Servant	36 (60.0%)	24 (40.0%)	
Self-employment	34 (64.2%)	19 (35.8%)	
**Number of people in a household**			
live alone	53 (65.4%)	28 (34.6%)	8.154 (0.043)
1-3	93 (56.7%)	71 (43.3%)	
4-6	11 (37.9%)	18 (62.1%)	
7-10	2 (100.0%)	0 (0.0%)	
**Socio-economic status**			
Low income	145(91.2%)	106(90.6%)	6.892(0.456)
High income	14(8.8%)	11(9.4%)	

### Factors associated with regular physical activity

The level of regular physical activity varied among the respondents. The results showed a majority of the participants were engaged in low (56.2%) levels of physical activity. A cross-tabulation was conducted between age, sex, marital status, religion, place of worship, ethnicity, occupation, work before retirement, minimum level of education, socioeconomic status, number of people in the household, and regular physical activity. The results show that age (χ² = 7.834, *p-value* = 0.005), minimum level of education (χ² = 4.368, *p-value* = 0.037), and number of people in the household (χ² = 8.154, *p-value* = 0.043) were statistically significant. There was no significant association between age, sex, marital status, religion, place of worship, ethnicity, occupation, work before retirement, socioeconomic status, and the level of regular physical activity. Two factors (age above 8 years and having a higher level of education) predicted older adults’ likelihood of participating in physical activity. The results showed higher odds of participating in physical activity among older adults who were aged 80 years (AOR = 4.740, 95%CI: 1.472–15.257 *p-value* = 0.009) compared to those 60 years The level of education significantly predicted a respondent’s likelihood of engaging in physical activity, especially for those with high education (AOR = 1.814, 95%CI: 1.024–3.212, *p-value* = 0.041). Table 3 shows the association between demographic characteristics and physical activity levels of older adults.

**Table 3:** Association between socio-demographic characteristics and physical activity.

	Level of Physical Activity		
Variables	Low	High	χ² (p-value)
**Age group**			
60-79	151 (97.4%)	108 (89.3%)	7.834 (0.005)
80-100	4(2.6%)	13 (10.7%)	
**Sex**			
Male	78(49.1%)	65(55.6%)	11.023(0.087)
Female	81(50.9%)	52(44.4%)	
**Marital status**			
Married	102(64.2%)	76(65.0%)	8.432(0.085)
Not married	57(35.8%)	41(35.0%)	
**Ethnicity**			
Ga, Akan	54(33.9%)	33(28.2%)	12.891(.0623)
Ewe,Mole Dagbani	105(66.1%)	84(71.8%)	
**Current employment status**			
Yes	41(25.8%)	39(33.3)	9.854(0.665)
No	118(74.2%)	78(66.7%)	
**Education level**			
Basic education	109(68.6%)	96(82.1%)	3.123(0.0876)
More than basic education	50(31.4%)	21((19.9%)	
**Religious Affiliations**			
Constructed places of worship	125 (80.6%)	106 (87.6%)	2.411(0.120)
Natural places of worship	30 (19.4%)	15 (12.4%)	
**Work Before Retirement**			
Not retired	39 (51.3%)	37 (48.7%)	2.443 (0.655)
Trader	25 (59.5%)	17 (40.5%)	
Farmer	25 (55.6%)	20 (44.4%)	
Not retired (Regularized post-retirement work)	36 (60.0%)	24 (40.0%)	
Self-employment	34 (64.2%)	19 (35.8%)	
**Socio-economic Status**			
Low to moderate income	143 (92.3%)	108 (89.3%)	0.743 (0.389)
High-income	12 (7.7%)	13 (10.7%)	

### Challenges of older adults in engaging in physical activity

The survey results highlighted a range of challenges and barriers that influence the participation of older adults in regular physical activity. Health-related issues (97.8%), lack of access to facilities and equipment (67.0%), and cultural perception (72.5%) were identified as important barriers to engaging in physical activity. The other factors were safety concerns in the neighborhood (88.4%), lack of personal motivation (64.5%), age-related stereotype (77.2%), bad weather conditions (50.4%), negative experience (72.8%), inadequate social support systems (67.0%), as important barriers to physical activity among older adults. However, the majority did not identify lack of time (46.1%), and cost (20.1%), as critical challenges older adults encounter in participating in regular physical activities with the community.

## Discussion

This study aims to identify factors that influence knowledge and regular physical activity among older adults in a peri-urban community. This aligns with previous findings that showed that regular physical activity tends to decline with age, with older adults being one of the most inactive segments of the population [15,29]. Regular physical activity is essential for managing and preventing hypertension among older adults, yet numerous factors, including inadequate knowledge levels, impede regular involvement. The relationship between knowledge levels and adoption is a critical component of physical activity behaviors among older adults. Many older adults do not have adequate knowledge of how physical activity can benefit the prevention of cardiovascular diseases [2,23]. The few participants with adequate knowledge could not identify the health benefits of regular physical activity, such as cardiovascular function, mental health, bone density, muscle strength, and the prevention of hypertension. Older adults must be conscious of educating older adults on the health benefits of regular physical activity [30,31]. The study draws attention to the fact that higher education is the foundational element in shaping the knowledge of older adults about physical activity and its health benefits. Using community-based workers for education in physical activity among older adults will help improve overall knowledge and increase motivation for adoption. This study demonstrated that older adults (56.2%) in peri-urban settings engage in physical activity for hypertension prevention. The decline in physical endurance, energy, and vitality could make it harder for older adults to engage in physical activities [32]. The World Health Organization – WHO (2022) postulated that physical activity encompasses various dimensions such as frequency, duration, intensity, types, context, and purpose. The types of activities reported by older adults in this study included walking and light household chores. Although these contribute to overall health, they often fall short in intensity and duration to achieve cardiovascular benefits [1]. However, physical activity for older adults must be prescribed in commensurate with their age, considering type, intensity, and frequency [33,34].

This study also identified a range of barriers that significantly influence both the knowledge and practice of physical activity among older adults. These barriers include health concerns, environmental challenges, and cultural beliefs. Even though knowledge is important, it is often not sufficient to drive behavior change, especially when practical barriers such as lack of resources or environmental constraints are present [2]. Intervention must go beyond education and must also address these practical barriers [2]. Many older adults expressed fears that engaging in physical activity could worsen their existing health conditions or lead to injury. Previous studies reported that older adults with chronic health conditions tend to have lower levels of physical activity due to concerns about exacerbating their conditions or experiencing injury [19,20,23]. This confirms the need for intensive adult education to increase awareness and prescription of older adults-specific physical activity. Environmental challenges and poor access to equipment and facilities were important in deterring physical activity among older adults. Individual health behaviors are influenced by multiple levels of factors, including the physical environment [35,36]. This reinforces the need for public health interventions that improve the physical environment, making it easier for older adults to engage in regular physical activity. The constructs influencing physical activity – knowledge, practices, and challenges are deeply interconnected and collectively influence the physical activity behaviors of older adults. By understanding and addressing these constructions, peri-urban communities can create a healthier, active population of older adults.

In terms of social influences, social support emerged as a significant motivator for physical activity, with spouses, family members, and community members often playing an active role in encouraging and facilitating physical activity [37,38]. This study found that older adults who had strong social networks, particularly those involved in religious activities, reported more engagement in physical activity. This is because one prominent barrier identified in this study is the influence of cultural misconceptions on physical activity, particularly those that discourage older adults from participating in regular physical activity. Cultural beliefs and traditional norms often dictate the roles and behaviors deemed appropriate for local community members [39,40]. These misconceptions stemmed from traditional views that associate physical activity with youthfulness and vitality, while older adults are envisaged to adopt a more sedentary lifestyle [39,40]. In peri-urban communities in Africa, religious institutions that serve as central community hubs could be effective platforms for promoting physical activity.

### Strengths and limitations

This cross-sectional study is one of the first to assess the factors associated with physical activity in the Dome community of the Ho municipality. The study sets the stage to begin to consider interventions to improve awareness and physical activity, especially among the aging population. Consequently, we assessed the barriers to these activities to encourage mitigating the same in developed interventions. However, some limitations ought to be acknowledged. The data collection was done by using a self-developed pretest questionnaire. Even though the test-retest reliability test produced an acceptable score, it might have some cultural influences in its construction. Another limitation was the inability to assess the participants’ ability to assess their knowledge of the range of activities they considered acceptable. The study was conducted within a specific context using cross-sectional methods with a convenient sampling technique; hence, generalization should be done with caution. Future studies must use experimental methods to test causal relationships and the level of influence of each factor on knowledge and adoption of physical activity among older adults. In addition, the data collection was done by trained research assistants (who were nurses and living in the same community) and could have been influenced by social desirability bias. Also, respondents were expected to recall previous daily activities to decipher those that could be regarded as physical activity. This, therefore, made the response prone to recall bias.

## Conclusion

This study provides a comprehensive understanding of the knowledge, practices, and identifies the complex and multifaceted barriers associated with physical activity among older adults. These barriers, ranging from individual and cultural misconceptions and environmental challenges to health concerns and motivational issues, are deeply rooted in the demographic, social, and economic realities in peri-urban communities. Understanding the factors that inhibit physical activity among older adults allows for the creation of tailored interventions. This will lead to the implementation of community-based health education programs focusing on the benefits of physical activity in preventing hypertension. Leveraging community health workers to spread information about the role of physical activity among older adults, especially in resource-limited settings, is imperative. Also, given that social support is an important motivator for physical activity, efforts should focus on enhancing social cohesion within local peri-urban communities. This could include group-based physical activities such as walking clubs or dance classes, which can foster social interaction. Local government authorities may also consider installing community-based machines, parks, and community centres to promote physical activity within neighborhoods.
